# The Impacts of the Perceived Transparency of Privacy Policies and Trust in Providers for Building Trust in Health Information Exchange: Empirical Study

**DOI:** 10.2196/14050

**Published:** 2019-11-26

**Authors:** Pouyan Esmaeilzadeh

**Affiliations:** 1 Department of Information Systems and Business Analytics College of Business Florida International University Miami, FL United States

**Keywords:** cognitive trust in competence, cognitive trust in integrity, emotional trust, perceived transparency of privacy policy, trust in health care providers

## Abstract

**Background:**

In the context of exchange technologies, such as health information exchange (HIE), existing technology acceptance theories should be expanded to consider not only the cognitive beliefs resulting in adoption behavior but also the affect provoked by the sharing nature of the technology.

**Objective:**

We aimed to study HIE adoption using a trust-centered model. Based on the Theory of Reasoned Action, the technology adoption literature, and the trust transfer mechanism, we theoretically explained and empirically tested the impacts of the perceived transparency of privacy policy and trust in health care providers on cognitive and emotional trust in an HIE. Moreover, we analyzed the effects of cognitive and emotional trust on the intention to opt in to the HIE and willingness to disclose health information.

**Methods:**

A Web-based survey was conducted using data from a sample of 493 individuals who were aware of the HIE through experiences with a (or multiple) provider(s) participating in an HIE network.

**Results:**

Structural Equation Modeling analysis results provided empirical support for the proposed model. Our findings indicated that when patients trust in health care providers, and they are aware of HIE security measures, HIE sharing procedures, and privacy terms, they feel more in control, more assured, and less at risk. Moreover, trust in providers has a significant moderating effect on building trust in HIE efforts (*P*<.05). Results also showed that patient trust in HIE may take the forms of opt-in intentions to HIE and patients’ willingness to disclose health information that are exchanged through the HIE (*P*<.001).

**Conclusions:**

The results of this research should be of interest to both academics and practitioners. The findings provide an in-depth dimension of the HIE privacy policy that should be addressed by the health care organizations to exchange personal health information in a secure and private manner. This study can contribute to trust transfer theory and enrich the literature on HIE efforts. Primary and secondary care providers can also identify how to leverage the benefit of patients’ trust and trust transfer process to promote HIE initiatives nationwide.

## Introduction

### Background

Trust plays a significant role in the situations where there is a distance between consumers and vendors, such as in internet-dependent contexts [1]. Health information exchange (HIE) networks share health information electronically with other care providers to improve care coordination and enhance patient safety. HIE projects can help primary and secondary health care providers by connecting them via information exchange networks. Different sharing mechanisms are being used by public and private health care organizations to facilitate information exchange initiatives [2]. Existing studies in HIE indicate that the following 3 exchange models are mainly applied by health care entities to electronically transmit patient health information: direct, query-based, and patient-centered exchange [3]. Electronic data exchange between providers can also take place at a regional or national level [4]. In a nationwide HIE project, health care organizations can exchange patients’ information across a huge network of providers consistent with nationally defined standards and contracts [5]. In a regional HIE initiative, medical records are shared electronically with unaffiliated hospitals or ambulatory providers in a particular region. A regional health information organization is a third party that enables information exchange across health care organizations within a community, county, or state HIE platform [6].

HIE initiatives utilize sharing mechanisms with which health information is mostly transmitted without a patient’s close supervision; thus, patient trust in the HIE is the core in this setting where a great deal of security concerns and privacy risks may be involved [3]. Trust in HIE technology can predict the direction of patients’ responses to the implementation of HIE in health care organizations, especially when patients perceive that they may not know everything about this sharing mechanism. The lack of awareness is mainly because of the distance imposed between patients and actual users (health care organizations), lack of direct interactions between patients and HIE networks, newness and evolving nature of HIE initiatives, and unfamiliar mechanisms used in the HIE system to share health information electronically. These characteristics create a setting that is more intangible than the traditional sharing methods (such as fax or mail). The mentioned reasons may make patient trust more critical in the HIE context.

At this point, the use of HIEs by patients or health care professionals is not at a stage of diffusion [7,8]. There is vigorous debate among the entities in the health care industry about the topic of opt-in versus opt-out of digital health records and electronic exchange of such information [9]. This debate argues whether health care providers or patients should have the right to decide whether the digital health information should be exchanged [10]. However, patients have the unconditional right to be aware of the data-handling practices of medical providers [11]. Public perspectives are important to researchers and policy makers because patients are one of the key stakeholders, and the widespread adoption of HIEs is not possible without their positive beliefs and attitudes toward this technology (such as trust factors). Therefore, it is noteworthy to determine whether consumers will choose to opt in to an HIE system if they are given the choice in the near future.

Human thoughts and decisions include cognition and emotion [12]; therefore, both beliefs and feelings should be investigated to better understand how a patient would trust and react to a system that is leveraged by other users (health care providers) to disseminate health information. Consistent with the trust transfer process [13], the level of trust in health care providers can be migrated to trust in HIE systems. Accordingly, patients’ trust in HIE characteristics can be derived from a trusted physician who has certain association with the HIE [14]. A patient has to trust an HIE system before he or she is willing to make an opt-in decision or disclose personal health information. Consumers will rely on a technology because of not only the technology’s efficiency and effectiveness but also the fair and honest relational exchange between the information technology (IT) and them [15]. Thus, trust can reflect its effects through a cognitive process (robust rational reasons) and an emotional procedure (strong affects and feelings).

In line with the previous research [16], individual trust is defined as the levels of trust in the specific characteristics of a trustee, such as competence, integrity, and benevolence. Extrapolating to the HIE context, it is expected that the patients should trust some HIE characteristics to opt in to the HIE and become more willing to disclose their personal health information. In the context of HIE, trust in competence refers to the trust in the HIE’s abilities, technical capabilities, skills, and expertise embedded in the technology. This dimension of trust implies the extent to which patients rely on technologically competent performance of HIE to effectively disseminate health information among a wide variety of health organizations. Trust in integrity describes the belief that the agreement between the patients and an HIE network is reliable, the HIE system honestly fulfills predetermined promises, and the HIE adheres to a set of principles that the patient finds acceptable. Trust in benevolence pertains to the belief that HIE cares about patients beyond the expected commitments to genuinely act in the patients’ interests. On the basis of this dimension, the HIE initiatives are believed to seek joint gain in dyadic relationships with patients aside from profit motives to openly follow patients’ welfare. Emotional trust implies an emotional security that enables individuals to feel assured that an IT will be responsive in uncertain situations.

Previous research on how patients’ trust in HIE is built is still scarce [17]. Despite the importance of patient trust in HIE, the nature of patient trust has not been thoroughly conceptualized, clearly measured, and fully delineated in this context. Previous studies mainly investigate different dimensions of cognitive trust (mostly trust in network design characteristics), and relatively little attention has been given to other trust dimensions [18]. Moreover, the difference between the different levels and dimensions of patient trust in the HIE context has not been analyzed. For a patient to trust an HIE network, the patient should feel assured that the HIE will not compromise personal health information and sensitive medical records and will not act unreasonably [19]. Sharing sensitive health information through a technology that is used by health care providers requires a new lens for understanding health consumers’ opt-in intention toward HIE. According to Kim et al [20], traditional IT research mostly focuses on organizational employees as users who adopt traditional IT for work-related purposes. In the contexts of many technology adoption studies, cognitive factors (eg, effort expectancy or facilitating conditions) can overshadow the effects of emotional variables (eg, emotional trust) on adoption decisions. The existing theories of IT adoption (such as technology acceptance model and unified theory of acceptance and use of technology) are mostly cognitive oriented and focus on users’ intention to accept and use a technology. However, in the HIE context, consumers are not the main users. Patients are the beneficiaries of HIE, but they are not the final users. The users are the health care professionals (ie, physicians and nurses), and the decision to adopt HIE is made at the practice or hospital level.

Information system (IS) literature shows that people feelings about IT impact their adoption decisions [21]. Our study is an attempt to extend this research stream by describing 2 aspects of trust (cognitive and emotional) and examining their roles in consumers’ opt-in intention and their willingness to disclose health information. The main point of this research is that when a technology (eg, an HIE) deals with sharing sensitive information and may exacerbate privacy concerns, patients will not only depend on cognitive factors to shape opt-in intentions and make information disclosure decisions. This study takes a trust-based perspective to investigate HIE adoption from the patients’ standpoint. On the basis of the study by Chopra and Wallace [22], trust plays an important role in situations where 2 sides are dependent, and this dependency may cause risk. In the context of HIE, given the amount of information exchanged among health care organizations, patients depend on HIE to improve treatment process, enhance care coordination, and increase the quality of care before they actually experience the possible effects. In this setting, risk can arise because patients may be concerned that too much personal information is shared, or erroneous health information is exchanged among health care providers through HIEs [23]. Therefore, health consumers’ reactions to HIE implementation largely depend on their trust in the HIEs.

To the best of our knowledge, the nature of trust and the differences between the dimensions of patient trust in HIE have not been clearly described. Few empirical studies examined the impact of trust in health care providers on building patient trust in HIE from a trust transfer mechanism. Moreover, patients’ decisions about HIE (such as opt-in decision) may not be purely cognitive based because of the special context in which this sharing technology is implemented and used. In many IT adoption decisions at the individual level, consumers’ affective reactions influence their choices [24]. In the HIE context, patients may not directly share their health information through exchange mechanisms, and they are distant from care providers who actually use these systems. Such a situation can downplay the pure impact of cognitive factors and give more weight to emotion because of the uncertainty associated with HIE and how this technology is used. Our study aims to advance the existing understanding of patient trust by defining and differentiating it in the HIE adoption setting from the patient’s perception. This study uses a balanced perspective to take both aspects of human experience (cognitive and emotional) into account and show whether emotional factors affect the consumers’ willingness to disclose health information and their intention to opt in to a technology designed to exchange their sensitive health information.

The purpose of the study was to contribute to the current literature in trust transfer and propose a practical solution to improving patient trust and opt-in rates for HIE. This study is conducted to contribute to the existing research by investigating how individual consumers develop trust in HIE and in what manner dimensions of trust will affect their resultant decisions related to HIE. This research is derived from the literature on trust transfer and IT adoption by articulating how perceived transparency of privacy policy and trust in health care providers impact opt-in intention and willingness to disclose health information through enhancing cognitive and emotional trust in HIE characteristics.

### Theoretical Background and Related Literature

Previous literature highlights the role of privacy statement in trust building in other contexts, for example, Web-based shopping, website registration, and mobile internet use. The completeness and transparency of Web privacy statements influence Web-based consumers’ perceptions and behavioral intentions to purchase products [25]. In electronic commerce (e-commerce) settings, the content of privacy statements is found as a significant factor to predict consumer trust in websites [26]. According to Callanan et al [27], user awareness of privacy policy has a direct effect on using mobile internet. The presence of a solid website privacy policy heightens the Web-based shoppers’ trust and, in turn, reduces their privacy concerns [28]. Framing a rigorous privacy statement that shows organizational compliance with the personal data protection regulations can significantly influence the consumers’ buying decisions [29].

As reported by Tsai et al [30], if Web-based retailers provide accessible and transparent privacy policy guidelines, consumers are more likely to pay a premium to purchase services from privacy protective websites. When a privacy statement is clearly presented by websites, consumers are more willing to read it carefully to get more Web-based services [31]. Recent studies indicate that adults are likely to avoid using mobile apps or opt out of Web-based services because of the absence of solid privacy statements [32]. If consumers are well informed about Web-based privacy terms and conditions, they provide more information to websites [33]. On the contrary, if no details are presented in privacy policies, customers are not aware of collecting and sharing procedures. Privacy policy dimensions contain details that empower customers by clarifying their rights and the options they may have to better control the use of health information. For instance, if they can opt out of information sharing with a third party, they will feel more control over their personal data, and this feeling makes Web-based services appear more trustworthy to them [34].

With the advance of technologies used for information exchange, a great number of consumers are anxious about the disclosure, transfer, and sale of personal information that organizations collect from them. Privacy policies should be framed to address patients’ privacy and security concerns. Privacy policy statements define how a health care organization collects, manages, uses, and disseminates personal health information (ranging from less sensitive to highly sensitive). Previous studies in the HIE context described that HIE privacy policies should be informative and comprehensive to reassure patients that exchanging their health information is a low-risk practice [35]. However, it is still not clear what type of contents, dimensions, and format an HIE privacy policy should cover to raise public awareness and build cognitive trust in HIE. Privacy policies are mainly devised based on the 5 dimensions of Fair Information Practice Principles: notice, access, choice, security, and enforcement [26]. Notice refers to the commitment of organizations to send timely announcements to consumers about their information collection practices before personal information is collected. Choice indicates that the consumers should be given the options about how the collected personal information would be used. Access means defining the consumers’ rights to view their own personal data and check whether such data are accurate and complete. Security defines the required steps and actions that should be taken by the organizations to ensure security and integrity of the consumers’ personal information. Enforcement articulates which national or international mechanisms, guidelines, and instruments are in place to enforce principles of privacy protection. Thus, HIE initiatives should clearly communicate their privacy policy standpoint to patients to increase the degree of trust.

A large number of IS studies treat trust as trusting beliefs [13,36]. Trusting beliefs are the cognitive beliefs shaped by the trustor based on the trustee’s trust-related characteristics (ie, competence, integrity, and benevolence) [12]. This cognitive trust is the result of a rational process in which a trustor expects that a trustee will own the required attributes that are reliable. Thus, cognitive trust is developed by a conscious calculation of advantages leading to rational reasons to trust a trustee. A mechanism that helps develop cognitive trust is the trust transfer, which is a cognitive process that may arise from a trusted entity to another new context [37]. According to Stewart [38], the trust transfer process relies on the relationships and interactions between the source and target. In the HIE settings, health care providers can be considered as the source, whereas the target is HIE systems, and the interaction is the efforts made to develop a transparent privacy policy model for information exchange. Thus, patients may form same perceptions about HIE because this technology will be used by the trusted health care providers. Nevertheless, rational expectations are not adequate for individuals to make trust-related decisions [39]. Previous trust literature describes trust in IT as a combination of both reasoning (cognitive trust) and feeling (emotional trust) [12]. Emotional trust, which is an individual’s evaluation of feeling and faith [40], is developed by emotional reactions to the trustee. In the context of dealing with an IT, emotional trust denotes whether an individual feels comfortable and secure about relying on the technology.

As cognitive and emotional trust are 2 different concepts, it is important to consider both types of trust in our research to portray a more comprehensive effect of trust on individuals’ reactions to the HIE implementation. On the basis of previous studies [41], trust in HIE is defined as follows:

#### Cognitive Trust in Competence

An individual’s rational beliefs about the technical expertise and ability of an HIE to exchange health information among health care entities.

#### Cognitive Trust in Integrity

An individual’s rational beliefs related to the honesty of the exchange process.

#### Cognitive Trust in Benevolence

An individual’s rational beliefs that an HIE system always considers the patient’s interest.

#### Emotional Trust

An individual’s feelings of assurance and security about relying on an HIE to share information across health care providers.

The 3 dimensions of trust are treated independently because they are conceptually and operationally different [13]. For instance, an HIE system may have the competence required to exchange information, but the consumers may be worried that the HIE might be designed to be biased by sharing sensitive information for other purposes (such as marketing). Alternatively, the consumers may perceive that an HIE network exhibits care to the patients, especially, in case new conditions of information sharing arise (when no agreement and commitment were made before), but the HIE does not have adequate technical capability. Trust in HIE’s benevolence is not easy to evaluate because individuals may not be likely to form the beliefs that HIE networks show care and goodwill beyond the main tasks of sharing personal health information in competent and honest manners. Previous studies in other contexts also indicate that cognitive trust in benevolence may not apply to every technology [39]. As there is no bilateral interactions and close personal relationships between patients and an HIE system, the HIE is not considered as a social actor, and cognitive trust in the benevolence of HIE may not be conceivable. Therefore, consistent with the key tasks HIEs are designed to perform, only cognitive trust in competence and integrity were used in this study.

The main theoretical foundation applied in this study was the Theory of Reasoned Action (TRA) [42]. According to TRA, an individual’s intention to perform a behavior is dependent on 2 variables: attitude and subjective norms. Attitude indicates an individual’s positive or negative feelings about a behavior, and a subjective norm denotes an individual’s perception about whether their significant others believe he or she should or should not engage in the behavior. Consistent with the study by Karahanna et al [43], the effect of subjective norms (which are normative beliefs) becomes more significant when there is a lack of experience with an IT. Furthermore, a subjective norm is a salient factor when a user perceives social pressure from important others to adopt a technology for his or her personal usage. In the context of HIE adoption, patients will not actually be able to use this technology and may only shape attitudes and form beliefs toward using a new system in health care organizations to manage information exchange among a wide range of providers [11]. Therefore, as the main objective of this study was to investigate the opt-in intention and information disclosure willingness of individuals who have experience with HIE, the proposed model focused on attitude and not subjective norms.

### Research Model

**Figure 1:**
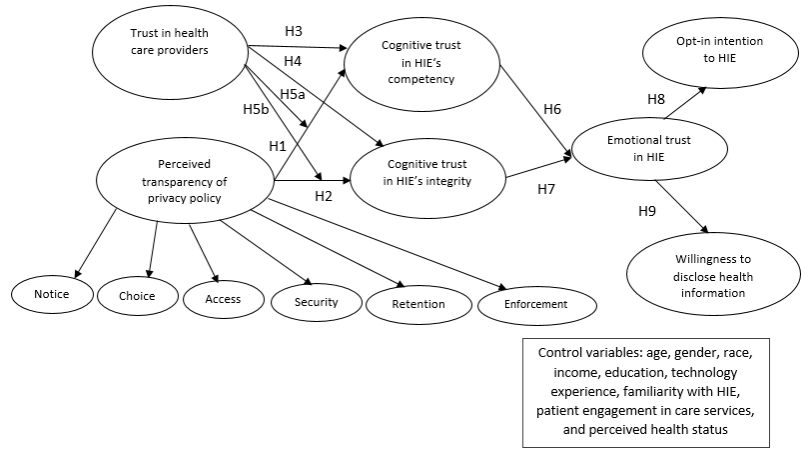
Research model. H: hypothesis; HIE: health information exchange.

The following research model (Figure 1), which is mainly based on a belief-attitude-intention framework, explains the causal relationships. The links begin with perceived transparency of privacy policy and trust in health care providers (perceptions) to cognitive trust (trusting beliefs) and emotional trust (trusting attitude), and finally ends with opt-in intention to HIE as well as willingness to distribute health information (trusting intention). In this study, we focused on the intention rather than the adoption behavior, as there is a solid evidence in the IS literature that shows that intention is a strong predictor of behavior [44].

In this model, cognitive trust in competence and integrity is considered as beliefs, and emotional trust is conceptualized as an attitude. Patients may believe that the HIE is competent and honest in sharing their health information based on firm rational reasons. Emotional trust plays the role of attitude toward the HIE adoption behavior, as it is an evaluative affect (ie, feeling secure) about trusting in HIE.

### Hypotheses Development

Consumers’ concerns in medical practices include high volume of collected health information, the possibility of privacy violations (eg, unauthorized access or hacked personal data), secondary use of medical records (eg, datamining purposes), lack of control over how medical records are collected, and how such information will be used [45-47]. Information privacy concerns may influence the validity and completeness of HIEs’ patient databases, which may result in wasteful investment, inaccurate treatments, erroneous care planning, and higher mortality rates [48]. To avoid such issues, HIE networks should assure patients that their medical records would be well protected. Privacy issues will influence consumer beliefs about HIE initiatives. The degree of trust between patients and HIE efforts may attenuate the information privacy concerns.

According to Dimitropoulos and Rizk [49], privacy concern is defined by the extent to which the health care entities (eg, providers and organizations) could access, view, and share patient health information without obtaining a permission or consent. A factor that may mitigate privacy concerns related to information exchange efforts and help form patient trust is the transparency of privacy policies. Thus, privacy policies should be clearly presented by the health care organizations to build patient trust in the HIE’s competence in protecting sensitive health information. The main objectives of the privacy policies are to enhance the understanding of how health information will be used inside or outside the organizations and decrease the concern that personal health information may be subject to improper access and would be used for unanticipated purposes [50]. The risk of information privacy misuse or unauthorized access highlights the importance of trust development before disclosing personal information. Previous studies emphasize that patients are concerned about losing control over the ways HIE systems handle their health information [51]. This concern mainly arises because of the lack of transparency of HIE information practices and policies. One of the best ways to address privacy concern and increase patient trust is through building a privacy policy with complete and transparent dimensions to clearly declare security tools and protection safeguards [27]. Comprehensible privacy policies should be developed by HIE initiatives to reduce the negative effects of information privacy concerns and improve patients’ cognitive trust in the HIE’s technical competence. The dimensions and principles included in the policies should be informative and transparent enough to be able to advance patients’ awareness of HIE data collection policies and information sharing practices. The more transparent the privacy policies are, the more they are likely to be reviewed and comprehended by patients, and only under this circumstance, patients are more willing to trust HIE’s technical abilities to protect health information.

Nowadays, patients are very likely to seek medical treatments and care services from different physicians and providers. HIE systems provide networks in which patients’ medical records are shared with a number of health care entities that are geographically scattered and use different privacy policies. In general, interoperable systems of data sharing between health care organizations are capable of improving completeness, reliability, and accuracy of medical records, which, in turn, ameliorate public health [52]. According to O'Kane et al [53], patients perceive that if the privacy policy is transparent, the electronic exchange of information among the health care providers is a more convenient and cost-effective sharing method, compared with the traditional data sharing efforts (eg, mail, phone, and fax transmission). With a clearly defined privacy policy, patients trust that a complete and flawless body of authorized information is shared electronically among health care entities through HIEs, and this is likely to help physicians generate better medical treatments and prescribe accurate medications. If privacy policy is perceived transparent, patients can learn about how health information is electronically shared between providers, what types of exchange mechanisms (eg, direct and look-up) are utilized to complete the sharing process, what types of sensitive information will be exchanged through the HIE, who will access and use the shared information, and for how long the information will be available to the authorized users. This perception may heighten trust in the HIE competence and encourage patients to believe that HIE technology is a real expert system in information-sharing area. Therefore, patients will become more familiar with HIE’s main functions and obtain a cognitive picture of the sharing procedures and security mechanisms associated with HIE. This cognitive map becomes a tool for them to facilitate their decisions to support the use of HIE by health care entities to improve care quality and reduce health care bills [54]. Therefore, the transparency of privacy policy dimensions is a sound and rational reason for the consumer to trust in the HIE’s competence. Accordingly, the following hypothesis is proposed:

H1: Perceived transparency of HIE’s privacy policy will positively influence cognitive trust in HIE’s competence.

Different industries have diverse levels of compliance because of the various degrees of confirmation requirements and the different levels of information sensitivity [55]. Organizations operating in the health care industry should satisfy a higher level of compliance because they deal with highly sensitive health information and medical reports. Thus, stricter policy guidelines are imposed on the industry sectors that process and handle highly sensitive personal information. HIE projects can take advantage of a transparent and accessible privacy policy to resolve concerns associated with data safety and potential misuse to win patient trust in HIE’s integrity, which, in turn, leads to competitive advantage. Privacy policies should be comprehensive and transparent enough to address all principles mentioned in the Health Insurance Portability and Accountability Act [49].

Notice principle articulates what health information is collected and exchanged, what the purpose of data exchange is, how such information will be used internally, and whether patient data will be disclosed to third parties. Choice principle delineates the consent process and permission requirements. This dimension provides options to patients regarding the use of their health data and the disclosure of such records to other third-party entities. For instance, by relying on this dimension, either patients are able to limit the exchange of personal information or voluntarily disclose their medical data for research purposes. Access principle entails granting the right to patients to obtain, review, and amend their personal information to ensure data accuracy and completeness. Security principle implies the adoption of reasonable measures and technical security steps to protect health information from unauthorized access, improper use, loss, unapproved alteration, or unanticipated disclosure during data exchange processes. Retention principle clarifies the acceptable duration of keeping and processing shared health information by health care providers. This dimension articulates the reasonable steps to permanently delete shared personal data if it is no longer required for the consented purpose. Enforcement principle highlights the self-regulation, such as privacy seals, that informs the public that the exchange procedures correspond to the legal requirements to protect information privacy [56]. Thus, highly transparent principles of privacy policies are able to demonstrate how safe, reliable, and dependent an HIE is and, in turn, increase patients’ cognitive trust in the HIE’s integrity.

The integrity of an HIE is the extent to which the HIE system is perceived to be honest and unbiased in the process of data sharing. However, an HIE system may be designed to adhere to a set of principles that are not acceptable by the patients. For instance, an HIE might collect, share, and use the patient’ personal information for purposes other than care provision without obtaining an authorization. Health information might have been shared with unauthorized entities for secondary use (such as marketing and research) [57]. Unauthorized third parties may illegally access patients’ sensitive medical records through HIE procedures and use such information for data mining purposes [18]. An HIE system with transparent privacy policy dimensions will be more effective in encouraging patients to trust a safe and credible mechanism that shares health information with authorized entities for legitimate purposes. Clear privacy policy dimensions attached to an HIE are likely to convince patients that the right amount of health information will be shared with authorized health care providers to meet relevant clinical purposes that are useful for patients’ treatments. Patients perceive that HIE systems that offer a more transparent privacy policy may tell the truth by fulfilling the agreed promises without any deviations. Thus, these HIE efforts would be better in line with consumers’ clinical preferences and heighten trust in integrity. An HIE privacy policy that is highly transparent to customers is perceived to be aligned with their health care–related expectations than any other party’s preferences. Compared with an HIE with low transparency, an HIE network with higher privacy transparency may be perceived to employ reliable procedures to grant access only to authorized users and apply honest exchange procedures to share health information for legitimate purposes. These reliable characteristics will increase the patients’ perceptions that the HIE’s procedures are unbiased.

According to Meinert et al [58], familiarity with the privacy policy statements can reduce the amount of risks and concerns related to an organization. Familiarity with the privacy policy of an HIE project can help patients develop a body of knowledge about what procedures are likely to be conducted and what mechanisms will be used in the future to exchange health information. This trust-related knowledge can increase the predictability power of patients to anticipate the HIE functions. If patients experienced some wrongdoing, dishonest procedures, deceptive information collection practices, unauthorized access, or illegal secondary use supported by an HIE system, they may predict that relying on the HIE systems is not wise. Consequently, they will think that the HIE network will also remain dishonest and untruthful in exchanging health information in the future. Thus, HIE’s transparent privacy policy will promote the patient’s trust in the HIE’s integrity.

H2: Perceived transparency of HIE’s privacy policy will positively influence cognitive trust in HIE’s integrity.

According to trust transfer theory, the trust transfer is a cognitive process in which the trust in one entity influences attitudes toward another phenomenon [59]. Trust transfer process describes that trust in a channel may affect the attitude toward a product or service offered in the same channel (intrachannel effects). Moreover, trust in a channel can be transferred to another channel because of perceived connections between them (interchannel effects) [37]. On the basis of the trust transfer theory, consumer trust in internet payment services may affect the level of consumer trust in mobile-based payment services [60]. In e-commerce settings, a study shows that trust can be transferred from the established and reputable websites to the unknown ones because of their links [38]. According to Lee et al [61], customers’ trust in an offline bank is transferred to its Web-based banking services and, in turn, influences perceived website satisfaction. Customers’ trust built over time in brick-and-mortar retailers is positively related to their level of trust in Web-based transactions before they visited their website [62].

As mentioned by Shin et al [63], trusted relationships between patients and providers play an important role in the acceptance of health informatics services. As HIE technology is mainly used by the health care providers to share personal information, trust in providers can form rational expectations that an HIE will also be a reliable and trustworthy sharing means [18]. Trusted interactions with the health care providers involved in the treatment process implies that the health care professionals will leverage a reliable, competent, and dependable mechanism for information exchange across organizations [64]. Heightened levels of trust in the providers can result in higher trust in the HIE’s technical capabilities and integrity because the patients may perceive that the providers will act in the best interest of patients with minimum risks [35]. Therefore, trust in health care providers may initiate a conscious calculation of HIE advantages by evaluating competency and reliability of exchange procedures that will be used to minimize privacy and security risks [65]. Consistent with the findings of the study by Tang et al [66], trusting relationships with providers lead to rational reasons to participate in information-sharing initiatives. If patients believe that they can rely on health care providers, they become more likely to reason that a sharing mechanism used by them is also reliable and competent [67].

In the context of our study, trust transfer can be a key factor in the HIE context where the transfer of consumers’ cognitive trust to the HIE’s competence and integrity will take place because of their trust accumulated over time in health care providers. On the basis of the provided discussions on the trust transfer process, we proposed that trust in reliable and dependable health care providers can positively affect patients’ cognitive trust in the HIE’s competence and integrity. Thus, we hypothesize the following:

H3: The level of trust a patient has in health care providers positively affects their cognitive trust in the HIE’s competence.

H4: The level of trust a patient has in health care providers positively influences their cognitive trust in the HIE’s integrity.

Trust building is a process of interactions between involved parties and technology [68]. According to Lu et al [60], the level of trust transferred from the internet to mobile payment services moderates the relationship between trust in mobile payment and customers’ behavioral intention. In the context of this study, we can argue that trust in health care providers also impacts the way privacy policy perceptions establish cognitive trust in the HIE’s competence and integrity. The level of trust in providers may change the direction of the path between perceived transparency of privacy policy and patients’ cognitive trust in the HIE. We proposed that the transparent privacy policy of the HIE initiatives can positively affect a patient’s cognitive trust in the HIE competence and integrity, but these relationships may be variable depending on the level of trust a patient has in health care providers. A transparent privacy policy presented by an HIE network may encourage patients to believe that the exchange project has required reliable characteristics to protect health information. However, the strength of privacy policy- cognitive trust link will change contingent on the levels of trusting relationship between patients and providers. Moreover, when poor trusting relationships are established with the health care providers in society, less transparency will be perceived from the HIE privacy policy, and patients will have less rational reasons to trust in the HIE. The privacy policy and cognitive trust relationship is improved when patients hold a trusting perception about health care providers. Thus, we propose that trusted interactions with health care providers reinforce the relationship between the perceived transparency of HIE privacy policy and the level of cognitive trust in the HIE. This helps us develop our next hypotheses as follows:

H5a: The level of trust a patient has in health care providers moderates the relationship between perceived transparency of privacy policy and cognitive trust in the HIE’s competence.

H5b: The level of trust a patient has in health care providers moderates the relationship between perceived transparency of privacy policy and cognitive trust in the HIE’s integrity.

Cognitive trust in the HIE is delineated by 2 dimensions: the rational expectations about the HIE’s ability to fulfil its obligations (cognitive trust in competence) and the rational reasons associated with the reliability of the HIE principles (cognitive trust in integrity). Emotional trust is defined as a patient’s comfort and security feelings about relying on the HIE to disseminate health information. Consistent with the findings of the study by Curtin et al [69], emotion is mostly evoked by cognition. A study by Komiak and Benbasat [39] highlights the positive relationship between cognitive and emotional trust. They suggest that if individuals analyze that a recommendation agent (such as a Web-based personalization technology) is logically reliable, they, in turn, become more likely to rely on it emotionally. Extrapolating from the previous studies to the HIE context, we can also argue that cognitive trust in the HIE’ competence and integrity is conceptualized as a belief. On the basis of the cognitive trust in competence, patients believe that the HIE is trustworthy because it has the required technological underpinning and competent exchange mechanisms to share health information among providers effectively and efficiently. Consistent with the cognitive trust in integrity, patients believe that the HIE is dependable for sharing health information because it holds reliable principles, truthful sharing standards, and honest promises. Consistent with TRA, these beliefs can strongly affect the attitude of patients toward the HIE efforts. Emotional trust is conceptualized as an attitude [42]. Emotional trust refers to an affective evaluation and feelings of relying on a trustee (such as a technology). In the context of HIE adoption, the higher the level of cognitive trust (both competence and integrity) in the HIE, the stronger the feelings of assurance, security, and comfort about the behavior of relying on the HIE. Therefore, the following hypotheses are developed:

H6: Cognitive trust in the HIE’s competence will positively influence emotional trust.

H7: Cognitive trust in the HIE’s integrity will positively influence emotional trust.

In this study, 2 related but different constructs are considered as dependent variables. Opt-in intention toward the HIE is the extent to which a patient is willing to rely on the HIE as a useful and reliable technology to be used by the health care entities to disseminate information. Willingness to disclose health information is the extent to which an individual is likely to share his or her sensitive health-related information with the health care organizations, with the knowledge that such information may be exposed to other providers through HIE systems. These 2 constructs are related because both of them are intention-based concepts; the first one is connected to adopting a technology (opt-in intention) and the second one is associated with a volunteer behavior (information disclosure). Nevertheless, they are different. The former variable deals with the notion that whether consumers are comfortable with the idea of having their health information shared through HIEs and whether to allow providers to use the system (if they are provided with the choice in the near future). The latter factor is the predictor of information disclosure behavior when the HIE systems are implemented by health care organizations. As patients typically cannot adopt an HIE, they can form attitudes, beliefs, and emotions about the concept of participating in sharing efforts. Therefore, in this context, the use should be evaluated through perceptual measures rather than actual opt-in behavior. A patient’s feelings of security and a strong sense of comfort about relying on an HIE network can increase the intention to opt in to the HIE system. Thus, emotional trust in the HIE can encourage patients to have their medical records shared with relevant entities.

Information disclosure intention indicates the willingness of the individuals to voluntarily reveal personal information about themselves to others [70]. Information disclosure intention has an important effect on sharing behaviors in different Web contexts (eg, e-commerce and Web-based health communities) [71]. In the HIE context, patients may be likely to disclose their information with providers participating in an HIE network in exchange for disease prevention, reduced health care costs, and more accurate and timely treatment suggestions. Previous studies highlight the importance of privacy and security concerns in the context of HIE implementation [49,54]. Patients will hold a positive attitude toward an HIE network when their health records are collected, stored, and exchanged confidentially [72]. According to Wright et al [73], if a patient’s privacy and security needs related to a data exchange mechanism are not met, he or she will become more likely to hide further health information from health care providers. Favorable attitude toward an HIE system is a result of a solid match between the HIE mechanisms and security or privacy requirements [3]. In this study, emotional trust is conceptualized as an attitude toward the HIE. In the presence of emotional trust, individuals are assured about the security of an HIE network and the privacy of their sensitive information that may be shared through this exchange means in the future. Thus, a high level of emotional trust in an HIE (ie, feeling secure about HIE use) will increase patients’ opt-in intention toward it. Moreover, we expect that patients holding a favorable attitude toward an HIE are more likely to disclose personal health information to providers using the HIE in their practice.

H8: Emotional trust will positively influence opt-in intention toward the HIE.

H9: Emotional trust will positively influence willingness to disclose health information.

Consistent with TRA, our model only proposes indirect relationships between perceptions (perceived transparency of privacy statement and trust in health care providers) and attitude (emotional trust) through beliefs (cognitive trust).

## Methods

### Measurement Development

This study drew on the existing literature to measure the constructs included in the model, and minor changes were made to the instrument to fit the HIE context. Items measuring opt-in behavioral intentions were adapted from the studies by Venkatesh et al [44] and Angst and Agarwal [11]. The scales used to measure cognitive trust in the HIE’s competency, cognitive trust in the HIE’s integrity, and emotional trust in the HIE were adapted from the studies conducted by Komiak and Benbasat [39] and Mpinganjira [41]. To measure the 6 dimensions of the perceived transparency of privacy policy (ie, notice, choice, access, security, retention, and enforcement), we adapted the items reported by Chua et al [56] and Wu et al [26]. In this study, the perceived transparency of privacy policy was measured as a reflective second-order construct with 6 dimensions. The rationale behind this measurement is that the perceived transparency is reflective of the 6 dimensions and the expected interactions among them. According to Kayhan [74], reflective modeling is a better option than formative when first-order factors are expected to interact, correlate, or share a common theme. Thus, interrelationships among these factors is an important component of measuring the perceived transparency. For instance, notice principle, which defines the purpose of data exchange and explains what information is shared, may be related to security dimension that defines the security safeguards used to protect such information and the data transmission process. To measure trust in health care providers, we adapted the items reported by Moon [65] and Gefen et al [36]. Finally, the items indicating willingness to disclose health information were adapted from Zhang et al [75].

Once the initial questionnaire was developed based on previous research, we used an expert judgment approach to enhance the content validity of the survey. To check for the completeness, accuracy, readability, and format of the survey, the questionnaire was sent to 7 experts who are well published in the field of health informatics and HIE. The content validity index testing was used to analyze the feedback and suggestions. In this approach, the team of experts indicated whether each item on a scale was congruent with (or relevant to) the construct. Then, the percentage of items deemed to be relevant for each expert was computed, and finally, the average of the percentages across experts was taken. The average congruency percentage (ACP) was 92, which was higher than the threshold of 90% [76]. Therefore, the ACP was considered acceptable for the survey used in this study. We then removed the marked ambiguous words and modified the questions based on the experts’ suggestions to ensure that they were clear and easy to understand for potential participants. Before conducting the main study, we conducted a pilot test with 137 graduate students at a large southeastern university in the United States to ensure the reliability and validity of the instrument. The Cronbach alpha was computed for each construct (perceived transparency of privacy statement, alpha=.96; trust in health care providers, alpha=.88; cognitive trust in the HIE’s competence, alpha=.85; cognitive trust in the HIE’s integrity, alpha=.90; emotional trust in the HIE, alpha=.91; opt-in intention toward the HIE, alpha=.92; and willingness to disclose health information, alpha=.92). All Cronbach alpha values were above the cutoff point of 0.7, which indicated that the instrument was internally consistent [77]. This study used 5-point Likert scales, with anchors ranging from 1=strongly disagree to 5=strongly agree. The final measure items used in this study are listed in Multimedia Appendix 1.

### Data Collection Procedure

Data were collected in June 2018 from Amazon's Mechanical Turk (MTurk) to obtain a representative group of subjects. As the HIE is still not considered as a routine technology for many individuals, to get more solid and reliable findings, we specified an additional qualification that individuals had to meet to participate in the survey. We defined a screening question to include only those individuals who had visited a health care provider participating in an HIE network. Thus, the participants were aware of the HIE efforts, and their health information was shared through an HIE project when they took part in this study’s data collection. The incentive for participation was a monetary reward (US $3). At the beginning of the Web-based survey, a detailed description of the HIE technology was provided to ensure that respondents completely comprehended the context and purpose of the study. The respondents were then asked a question about their level of familiarity with HIEs. To capture the dynamic trust transfer process and double check on whether their experience with the HIE projects met our criteria, before answering the main survey questions, they were requested to describe why and how they were familiar with HIEs. In total, 517 individuals attempted the survey. The respondents’ answers to the familiarity question were analyzed to detect the main reasons they were aware of the HIE. Almost 94.9% (491/517) of the respondents were familiar with HIEs through *visiting a (or multiple) doctor who participated in an HIE network*. The remaining 5.0% (26/517) were aware of HIEs because of other reasons such as *through the internet searching/social media*, *reading health care magazines/newspaper*, *friends/family*, and *working in health care*. As we only focused on individuals who were familiar with HIEs because of visiting providers that actually shared their information through HIE networks, 16 potential participants were discarded and 501 met this condition.

As mentioned in previous studies, a general concern in data collection is the potential lack of attention and random responses [78]. Consistent with other studies, we used *captcha* questions to prevent and identify careless, hurried, or haphazard answers [79]. On the basis of the answers to these questions, 8 responses were dropped. This ratio is similar to those reported in previous studies that used MTurk for data collection [80]. Thus, concerns that Web-based respondents might reply randomly or haphazardly to complete the survey quickly were alleviated. After excluding responses that failed the response quality questions, the final set of usable and valid responses contained 493 samples. Moreover, the average completion time was 15.3 min that given the number of questions in the survey, suggested respondents spent an acceptable amount of time completing it.

Then, participants were requested to complete the survey by answering questions regarding the last time a health care provider used an HIE network to share their health information with other entities (such as other hospitals, physician practices, laboratories, pharmacies, primary care, and emergency department). To ensure that their experience was recent enough, and so, they were able to remember its details, they were asked to indicate how many times they visited a (or multiple) doctor participating in an HIE project and when the most recent one was. Respondents had visited a (or multiple) physician involved in an HIE effort an average of 4.32 times during the previous year, and the most recent experience ranged from 2 months to a week ago. Relying on these screening questions and figures, the final sample fitted the study objective, which was investigating the trust of individuals (who were experienced with an HIE through providers who shared their records using the HIE) in the HIE and their opt-in intention toward it.

When testing the research model in this study, we controlled for consumer demographics and contextual factors such as income, age, education, race, gender, general technology experience, perceived health status, and engagement in the health care service, which are found and tested by the previous research as important factors in the adoption of HIEs. Therefore, it could be argued that by controlling the effects of aforementioned variables, the opt-in intention toward the HIE and willingness to disclose health information will mainly be measured based on the elements of cognitive and emotional processes linked with the health consumers’ beliefs and attitudes toward electronic data exchange.

### Instrument Validation

To validate the survey instrument, we performed confirmatory factor analysis on all the constructs to assess the measurement model. To do so, International Business Machines Corporation SPSS Amos (version 22) was used to test convergent and discriminant validity. According to Gefen et al [81], convergent validity can be tested by examining the standardized factor loading, composite reliability, and the average variance extracted (AVE). Table 1 shows the results of convergent validity test. All values of composite reliabilities were more than the threshold value of 0.7, which highlighted that the reliability of constructs was adequate [82]. According to Hair et al [83], a factor loading of ≥0.7 is acceptable. In this study, all reported standardized factor loadings were >0.7. The AVE of each construct was calculated using standardized factor loadings. All reported values of the AVE were also >0.5, which met the minimum requirement [84]. These measures indicated that the convergent validity of the measurement model was acceptable.

We also tested the discriminant validity of the constructs (Table 2). All the diagonal values were >0.7 and exceeded the correlations between any pair of constructs [85]. Therefore, the result indicates that the model fulfills the requirements of discriminant validity, and we can assume that the model also has adequate discriminant validity.

Although the correlations among constructs were not very noticeable (eg, a correlation of 0.483 between cognitive trust in the HIE’s competence and integrity), we checked for multicollinearity by computing the variance inflation factor (VIF) and tolerance values for the predictor variables. The resultant VIF values were between 1.385 and 1.831, which were below the cutoff value of 5, and the tolerance values were in the range of 0.546 and 0.722, which were greater than the threshold of 0.1 [77]. Thus, the multicollinearity is not an issue in this research.

**Table 1:** Results of convergent validity.

Construct and respective items			Standardized factor loading (>0.7)	Composite reliability (>0.7)	Average variance extracted (>0.5)
**Perceived transparency of privacy policy**					
	**Notice**				
		1	0.81	0.927	0.716
		2	0.85	—^a^	—
		3	0.86	—	—
		4	0.87	—	—
		5	0.84	—	—
	**Choice**				
		1	0.83	0.92	0.696
		2	0.83	—	—
		3	0.85	—	—
		4	0.86	—	—
		5	0.8	—	—
	**Access**				
		1	0.81	0.884	0.657
		2	0.81	—	—
		3	0.77	—	—
		4	0.85	—	—
	**Security**				
		1	0.81	0.913	0.723
		2	0.86	—	—
		3	0.87	—	—
		4	0.86	—	—
	**Retention**				
		1	0.83	0.916	0.731
		2	0.87	—	—
		3	0.84	—	—
		4	0.88	—	—
	**Enforcement**				
		1	0.88	0.903	0.757
		2	0.87	—	—
		3	0.86	—	—
**Trust in health care providers**					
	1		0.81	0.872	0.695
	2		0.84	—	—
	3		0.85	—	—
**Cognitive trust in the competency of health information exchange**					
	1		0.75	0.875	0.638
	2		0.81	—	—
	3		0.86	—	—
	4		0.77	—	—
**Cognitive trust in the integrity of health information exchange**					
	1		0.82	0.916	0.687
	2		0.86	—	—
	3		0.86	—	—
	4		0.83	—	—
	5		0.77	—	—
**Emotional trust in health information exchange**					
	1		0.87	0.932	0.775
	2		0.88	—	—
	3		0.9	—	—
	4		0.87	—	—
**Opt-in intention to health information exchange**					
	1		0.81	0.926	0.758
	2		0.89	—	—
	3		0.88	—	—
	4		0.9	—	—
**Willingness to disclose health information**					
	1		0.89	0.929	0.766
	2		0.83	—	—
	3		0.9	—	—
	4		0.88	—	—

^a^Not applicable.

**Table 2:** Results of discriminant validity (the main diagonal elements in italics denote the square roots of the average variances extracted, and the off-diagonal values represent the correlation coefficients between the constructs).

Construct	NOT^a^	CHO^b^	ACC^c^	SEC^d^	RET^e^	ENF^f^	THP^g^	CTC^h^	CTI^i^	EMT^j^	INT^k^	WILL^l^
NOT	*0.846*		—	—	—	—	—	—	—	—	—	—
CHO	*0.372*	0.*834*	—	—	—	—	—	—	—	—	—	—
ACC	*0.421*	0.479	0.*810*	—	—	—	—	—	—	—	—	—
SEC	*0.467*	0.421	0.447	0.*850*	—	—	—	—	—	—	—	—
RET	*0.358*	0.393	0.459	0.464	0.*854*	—	—	—	—	—	—	—
ENF	*0.411*	0.373	0.387	0.378	3.764	0.*870*	—	—	—	—	—	—
THP	0.381	0.323	0.515	0.446	3.862	0.485	*0.833*	—	—	—	—	—
CTC	0.396	0.356	0.496	0.399	3.898	0.454	0.317	*0.798*	—	—	—	—
CTI	0.367	0.464	0.322	0.323	3.670	0.517	0.396	0.483	*0.828*	—	—	—
EMT	0.434	0.378	0.505	0.356	4.254	0.523	0.261	0.358	0.474	*0.880*	—	—
INT	0.418	0.446	0.478	0.229	3.941	0.545	0.368	0.335	0.382	0.324	*0.870*	—
WILL	0.359	0.399	0.497	0.315	0.553	0.505	0.372	0.380	0.375	0.306	0.490	*0.875*

^a^NOT: notice.

^b^CHO: choice.

^c^ACC: access.

^d^SEC: security.

^e^RET: retention.

^f^ENF: enforcement.

^g^THP: trust in health care providers.

^h^CTC: cognitive trust in the competency of health information exchange.

^i^CTI: cognitive trust in the integrity of health information exchange.

^j^EMT: emotional trust in health information exchange.

^k^INT: opt-in intention to health information exchange.

^l^WILL: willingness to disclose health information.

## Results

### Descriptive Statistics

Table 3 depicts respondents’ characteristics. The demographic characteristics show that the majority of the respondents were male (272/493, 55.1%), white (369/493, 74.8%), with a full-time job (338/493, 68.6%), and had a Bachelor’s degree (257/493, 52.2%). Over 70% of respondents were aged between 20 and 39 years, and around 28% of the sample was aged >40 years.

Our sample could be a representative of the actual demographics of the HIE users, as this is consistent with the age distribution in previous studies on the HIE [54,86].

Respondents of this study were fairly familiar with the general (eg, the internet and computer) and health care (eg, health tracking apps, Web-based patient community, and personal health record) technologies. Moreover, they were healthy enough to participate in the Web-based survey and were relatively engaged in their own care. Table 4 shows characteristics such as respondents’ technology background, health status, and levels of engagement in care. The descriptive statistics of constructs used in the conceptual model are shown in Table 5.

**Table 3:** Sample (N=493) characteristics.

Variable and respective categories		Percentage
**Gender**		
	Male	55.1
	Female	44.9
**Age (years)**		
	<20	0.4
	20-29	35.8
	30-39	35.8
	40-49	13.3
	50-59	8
	≥60	6.6
**Annual household income (US $)**		
	<25,000	13.3
	25,000-49,999	32.3
	50,000-74,999	24.3
	75,000-99,999	16.4
	≥100,000	13.7
**Education**		
	Less than high school	1.8
	High school graduate	12.8
	Some college	19.5
	2-year degree	8.4
	Bachelor’s degree	52.2
	Graduate degree	5.3
**Employment status**		
	Employed—full time	68.6
	Employed—part time	16.8
	Unemployed	6.2
	Retired	5.3
	Student	3.1
**Race or ethnicity**		
	White	74.8
	African American	8.4
	Asian	9.7
	Hispanic	4.9
	Mixed	2.2
**Participation in a Web-based patient community**		
	Yes	52.2
	No	47.8
**Using a personal health record**		
	Yes	63.7
	No	36.3

**Table 4:** Sample technology background, engagement level, and health status.

Variable	Descriptive statistics, mean score (SD)
Perceived health status	3.98 (0.783)
Computer skills	4.41 (0.668)
Comfortable with using computers	4.61 (0.680)
Comfortable with using the internet	4.69 (0.581)
Comfortable with using mobile devices or apps for health purposes	4.33 (0.947)
Patient commitment	3.72 (0.68)
Therapeutic alliance	3.48 (0.79)

**Table 5:** Descriptive statistics of constructs (all measures are 5-point scales, with anchors 1=strongly disagree and 5=strongly agree).

Constructs	Descriptive statistics	
	Mean (SD)	Variance
Notice	3.75 (0.97)	0.94
Choice	3.62 (0.99)	0.99
Access	3.53 (0.95)	0.91
Security	3.62 (0.99)	0.99
Retention	3.51 (1)	1
Enforcement	3.64 (1.05)	1.11
Trust in health care providers	3.76 (1.01)	1.02
Cognitive trust in HIE’s^a^ competency	3.64 (1.05)	1.11
Cognitive trust in HIE’s integrity	3.75 (0.97)	0.94
Emotional trust in HIE	3.62 (0.99)	0.99
Opt-in intention to HIE	3.64 (1.05)	1.11
Willingness to disclose health information	3.62 (1.05)	1.11

^a^HIE: health information exchange.

### Control Variables

Factors that do not represent the core variables (ie, those included in the causal model) of this study, but which nevertheless may affect the interrelationships between the core variables, have been controlled for. These factors include age, gender, race, income, education, technology experience, familiarity with HIE, engagement in the care services, and perceived health status. Although the causal model seems to represent consumers’ opt-in intention and determine their willingness to disclose health information, we found that the effects of control variables were not negligible. On the basis of the results, 2 dimensions of patient engagement in care (ie, patient commitment and therapeutic alliance) [87] directly influence both the HIE opt-in intention (beta=.204; *P*<.01) and disclosure willingness (beta=.127; *P*<.05) as contextual factors. This implies that factors that drive the patients to seek a greater understanding of their conditions will encourage them to opt in to the HIE and disclose health information. Moreover, the levels of patient’s connection to the providers in the pursuit of care goals influence the trust building process and opt-in decision making. The findings also show that age (beta=−.141; *P*<.01), education level (beta=.112; *P*<.05), technology experience (beta=.132; *P*<.01), and HIE familiarity (beta=.247; *P*<.001) influence opt-in intention toward the HIE. These effects indicate that younger patients who are more familiar with the HIE networks and also have higher educational and technology experience backgrounds may have higher intentions toward the implementation of the HIE. Among the control variables, only education level affects the willingness to disclose health information (beta=.186; *P*<.01), meaning, individuals with higher levels of education are more likely to share their personal health information with providers. In contrast, no effects of gender, race, income, and perceived health status were found on both opt-in intention and willingness to disclose health data.

### Structural Model

International Business Machines Corporation SPSS Amos (Version 22) was used to test the hypotheses within a structural equation modeling [88] framework. According to Ho [89], the goodness of fit statistics can evaluate the entire structural model and assess the overall fit. The findings indicated that the value of chi-square divided by degree of freedom for the model was X^2^/df=3507.2/1563=2.2. The index values for confirmatory fit (0.914), normed fit (0.921), relative fit (0.923), and Tucker-Lewis (0.936) indices were above 0.9 and the standardized root mean square residual (0.035) and root mean square error of approximation (0.047) were below 0.08 [90]. All these measures of fit were in the acceptable range, and only goodness of fit index (GFI; 0.851) and adjusted GFI (0.822) were marginal. On the basis of the study by Kline [91], at least 4 of the statistical values met the minimum recommended values, which supported a good fit between the hypothesized model and the observed data.

**Figure 2:**
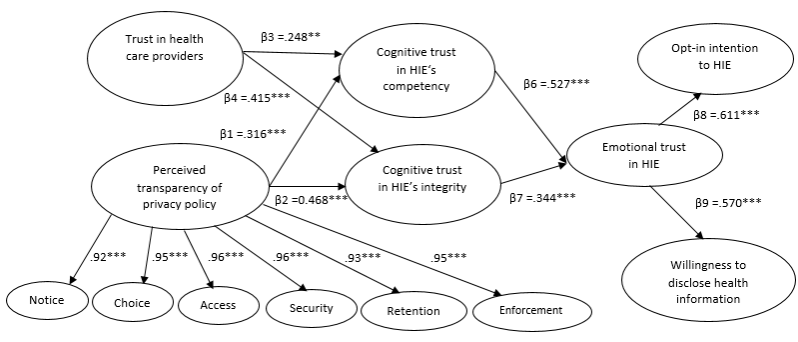
Model paths (**P<.01; ***P<.001). β: beta value; HIE: health information exchange.

The results show that the perceived transparency of privacy policy is more accurately modeled and measured in the context of HIE as a second-order construct with 6 factors (ie, notice, choice, access, security, retention, and enforcement). The expectation of interactions is confirmed by the presence of significant positive correlations between the 6 dimensions. Moreover, the path values of the 6 indicators (notice: 0.92; choice: 0.95; access: 0.96; security: 0.96; retention: 0.93; and enforcement: 0.95) are significant (*P*<.001). Figure 2 displays the standardized path coefficients of the structural model under investigation and depicts the significant predictors of patients’ opt-in intentions toward HIE and willingness to share health information.

The results of hypotheses testing are summarized in Table 6. The findings provide enough evidence to support H1, which indicates that the perceived transparency of privacy policy significantly increases cognitive trust in the HIE’s competence (beta=.31; *P*<.01). The analysis also demonstrates that the perceived transparency of privacy policy is a significant antecedent of cognitive trust in the HIE’s integrity (beta=.46; *P*<.001), and this positive linkage supports H2. Moreover, the R^2^ scores for the 2 types of cognitive trust are 0.49 (cognitive trust competency) and 0.58 (cognitive trust in integrity), respectively. The results support H3 by showing the significant positive relationship between the trust in health care providers and the cognitive trust in the HIE’s competence (beta=.24; *P*<.01). H4 is also supported where the higher level of trust in health care providers leads to higher cognitive trust in the HIE’s integrity (beta=.41; *P*<.001). H6 argues the existence of a positive relationship between cognitive trust in the HIE’s competence and emotional trust in the HIE, which is supported by the statistics (beta=.52; *P*<.001). Support is also found for H7, with cognitive trust in the HIE’s integrity significantly affecting emotional trust in the HIE (beta=.34; *P*<.01). Furthermore, the R^2^ score for emotional trust is 0.48. The findings provide solid evidence to support H8 by indicating that the higher the emotional trust in the HIE, the more likely patients are to allow health care providers to electronically exchange their health information using the HIE networks (beta=.61; *P*<.001). In addition, the positive effect of emotional trust to entice patients to disclose their health information is significant, supporting H9 (beta=.57; *P*<.001). Finally, the R^2^ scores for opt-in intention and willingness to disclose health information are 0.56 and 0.51, respectively, reflecting that the model provides relatively strong explanatory power to predict the variance in the patients’ willingness to release their health data and their intentions to opt in to HIE systems.

**Table 6:** Results of hypotheses testing (all results supported the hypotheses).

Hypothesis	Path	Standardized coefficient	SE	Critical ratio
H1	PTPP^a^ to CTC^b^	0.316^c^	0.077	11.265
H2	PTPP to CTI^d^	0.468^e^	0.074	11.152
H3	THP^f^ to CTC	0.248^c^	0.063	4.649
H4	THP to CTI	0.415^e^	0.057	6.991
H6	CTC to EMT^g^	0.527^e^	0.066	6.806
H7	CTI to EMT	0.344^c^	0.082	8.195
H8	EMT to opt-in intention to health information exchange^h^	0.611^e^	0.062	12.669
H9	EMT to willingness to disclose health information^i^	0.570^e^	0.063	13.579

^a^PTPP: perceived transparency of privacy policy.

^b^CTC: cognitive trust in the competency of health information exchange (R^2^=0.49).

^c^*P*<.01

^d^CTI: cognitive trust in the integrity of health information exchange (R^2^=0.58).

^e^*P*<.001

^f^THP: trust in health care provider.

^g^EMT: emotional trust in health information exchange (R^2^=0.48).

^h^R^2^=0.56

^i^R^2^=0.51

### Moderating Effect of Trust in Health Care Providers

The moderating effect of trust in health care providers on the paths of perceived transparency of privacy policy to cognitive trust in the HIE’s competence and cognitive trust in the HIE’s integrity are significant at .05. To further interpret the interactions, separate regression analyses were conducted for subgroups of the sample. According to the approach of 1 standard deviation below and above the mean [89], the sample was split into 2 subgroups: low provider trust and high provider trust. Then, the relationship between perceived transparency of privacy policy and cognitive trust in the HIE’s competence was regressed for each subgroup. The same analysis was conducted on the path of perceived transparency of privacy policy to cognitive trust in the HIE’s integrity. The moderating test results indicate that the positive relationship between perceived transparency of privacy policy and cognitive trust in the HIE’s competence is stronger among those who have well-established trusting relationships with health care providers. The findings also reveal that there is a significant difference in the relationship between perceived transparency of privacy policy and cognitive trust in the HIE’s integrity between high versus low provider trust subgroups (critical ratio of difference between the 2 groups=2.51; *P*<.05). This finding supports our hypotheses (ie, H5a and H5b) that indicate that the relationship between perceived transparency of privacy policy and cognitive trust in the HIE’s competence and the linkage of perceived transparency of privacy policy with cognitive trust in the HIE’s integrity are positively moderated by the trust level in health care providers.

## Discussion

### Principal Findings and Implications for Research

The main findings of this study indicate that trust in health care providers and perceived transparency of privacy policy are significant predictors of cognitive trust in the HIE’s competence and integrity. Our study also shows that the levels of trust in health care providers can moderate the relationships between perceived transparency of privacy policy and dimensions of cognitive trust in the HIE. Therefore, trusting relationships between patients and providers can strengthen the effects of privacy policy on cognitive trust in the HIE networks. Moreover, the results demonstrate that cognitive trust in the HIE can improve emotional trust in the HIE projects. Finally, emotional trust in the HIE efforts can encourage patients to opt in to HIE projects and become more willing to disclose their personal health information.

This research contributes several implications for theory. First, as trust has been proposed by previous studies as an important variable in the context of HIE rollout [92], it is crucial to add, and empirically test, different dimensions of trust in the HIE adoption research stream. In this study, the cognitive trust in competence, cognitive trust in integrity, and emotional trust are separated to provide a more complete evaluation of trust effects on patients’ intentions to endorse the use of HIE and its possible impacts on their information disclosure decisions. In the context of HIE, previous studies mostly consider trust as trusting beliefs [18,35]. To offer more comprehensive insights, both cognitive and emotional dimensions of patient trust in the HIE are investigated. Our proposed model posits that cognitive trust is reflected as beliefs and emotional trust is conceptualized as attitude. In line with TRA and previous studies that use this theory in other contexts, the findings show that cognitive trust in the HIE’s competence and integrity (beliefs) significantly influences emotional trust in the HIE (attitude). This is consistent with psychology studies [69] suggesting that emotion is triggered by cognition, and it directly influences decision-making process.

This study differentiates between cognitive and emotional trust to contribute to the understanding of the process of patient trust formation in the HIE context. Cognitive trust in HIE is delineated by 2 dimensions: the rational expectations about the HIE’s ability to fulfill its obligations (cognitive trust in competence) and the rational reasons associated with the reliability of the HIE principles (cognitive trust in integrity). Emotional trust is defined as a patient’s comfort and security feelings about relying on the HIE to disseminate health information. This study also indicates that perceived transparency of privacy policy is able to resolve uncertainty associated with information-sharing processes, advance patient awareness of the HIE, and generate knowledge about how it operates. Then, patients’ interpretation of their knowledge will directly affect cognitive trust in competence and integrity. In line with the findings of the study by Kahn et al [93], rational expectations and reasons that the HIE has the necessary characteristics to be relied upon will affect the degree to which patients feel in control, secure, and comfortable (emotional trust) about relying on the HIE to share their personal health information. On the basis of the significant relationships described in the model, when a patient is cognitively and emotionally involved with the HIE system, and trust is formed, he or she becomes more likely to disclose health information and allow health care providers to leverage this technology to share such information electronically with other health care parties.

Second, we draw upon the trust transfer theory to explain how patients’ trusting beliefs in the HIE are formed in the context of HIE. This study examines trust transfer as a salient means of establishing initial trust in the HIE initiatives. The model developed in this study highlights how trust in health care providers (as the main users of HIE) is migrated to patients’ cognitive and emotional trust in the HIE and how these trust factors will influence patients’ opt-in decisions and their willingness to share personal health information. The results are consistent with the findings of the study by Lin et al [37], indicating that before a consumer accepts a technology, his or her past encounters and experiences may influence his or her beliefs about the new technology. The significant impact of trust in health care providers on the cognitive trust levels in the HIE’s competence and integrity provides an empirical evidence on a dynamic of trust transfer process between health care professionals and health-related technologies (such as the HIE systems). This implies that well-established trust in health care providers strongly transfers to patients’ cognitive trust in a technology designed to share sensitive health information across health care entities. Visiting reliable and responsible health care providers can lead to a rational process in which a patient expects that an HIE network is designed in a way to own the required features that are dependable. The significant interaction relationships between trust in health care providers and cognitive trust in the HIE’s competence and integrity further validate the important role of previous experience with trusted providers in establishing positive beliefs and attitudes toward the HIE initiatives. The proposed model also highlights the importance of trust transfer mechanism in building cognitive trust in the HIE, emotional trust in the HIE, adoption behavior of patients, and their willingness to share their health information in the future.

Third, we figure out 3 factors to understand the trust transfer process: trust in source (trust in health care providers), trust in target (trust in the HIE), and the relationships between the source and the target (formulating a transparent privacy policy). According to the model, we propose that the trust in health care providers and the interactions between health care providers and the HIE (through the development of a transparent privacy policy to protect health information) can affect patient trust-building process. As patient trust in the HIE has become a critical factor for most of the HIE networks [94], the research on how this phenomenon is formed is of critical value. Our study delivers a comprehensive picture of the trust transfer process by highlighting the role of trust in source (providers), trust in target (HIE initiatives), and the interactions between source and target through creating a solid and comprehensive privacy policy for information exchange across various providers. In a study by Delgado-Ballester et al [95], perceived business tie is considered as the main predictor of trust in target. In contrast, Lin et al [37] argue that trust in source is the only key factor in the trust transfer process. Our study is an attempt to provide a full picture of trust transfer mechanism in the HIE context by indicating that both trust in health providers and the relationships between the providers and HIE can impact patients’ trust in the HIE systems.

Our research identifies a factor to capture the interactions between the providers and HIE efforts. The perceived transparency of privacy policy is considered as the factor reflecting the tie and linkage between the providers and HIE networks. This enriches the trust transfer literature by showing that not only initial trust in providers can play an important role in developing patient trust in the HIE but also the beliefs that trusted providers will make a significant contribution to the development of a comprehensive and transparent privacy policy will meaningfully affect the patient trust-building process. This finding also implies that trust in health care providers has both direct and moderating effects on the cognitive trust in the HIE’s competence and integrity. Relying on the moderating effect proposed in the model, our findings also answer the following question: will patients always trust an HIE system if a robust privacy policy with transparent components is provided and reliable security safeguards are leveraged to protect the health data in the information transmissions? The strong moderating effect of trust in health care providers on the relationships between the perceived transparency of privacy policy and the level of cognitive trust in the HIE’s competence and integrity can address this question. This study provides empirical evidence that the greater levels of trust patients have in health care providers can reinforce their perceptions that a highly transparent and solid privacy policy is attached to the HIE initiatives, and, in turn, their cognitive trust in the HIE will be improved.

Fourth, the results show that trust transfer factors have explained more than half of the variance in opt-in intention toward the HIE and individuals’ willingness to disclose health information. Therefore, we can predict that trust transfer process is a strong explanatory mechanism to understand how patients’ trust in the HIE efforts is built. We also believe that our model is not necessarily limited to the HIE but would be applicable to other technologies in the health care industry with similar characteristics, such as electronic health record and electronic prescribing systems. Finally, this study enriches the HIE literature by applying trust transfer theory to this research domain. Different from the traditional exchange mechanisms (such as mail or fax), the development of the HIE networks has largely pushed the information sharing among health care providers from conventional approaches to electronic exchange mechanisms. As patients are not the main users of the HIE systems and because of the distance imposed between patients and the actual users (providers), patients’ past perceptions about health care providers may be transferred to the electronic exchange context. This study uses the trust transfer perspective to explain the theorization of the HIE adoption by capturing the dynamics of trust-building process.

### Implications for Practice

There are also a number of important practical implications derived from this study. First, the significant role of trust in health care providers to predict cognitive trust in the HIE and the moderating effect of trust in providers in the relationship between privacy policy development and cognitive trust suggest that health care providers with good reputation can practically advance patient engagement in the HIE efforts. In contrast, patients are not likely to support and participate in the HIE efforts if these systems are developed or managed by providers with relatively poor reputation because of previous data breaches. Consistent with the findings of the study by Lu et al [60], consumers’ initial lack of trust in health care providers can become a significant barrier in the implementation of the HIE projects. Thus, participations of trusted providers in the implementation of the HIE initiatives and contributions of reputable health care organizations to the development of comprehensive and transparent privacy policies should be highlighted in the HIE projects to win the cognitive and emotional trust of patients. Patient trust can be used as an enabler that allows a health care provider to expand from the traditional sharing methods to the HIE models (such as direct exchange or query-based exchange) [96]. Health care providers should look for opportunities to nurture their patients’ trust in projects designed to exchange health information electronically. They should consider using tactics to increase the transparency and completeness of the HIE privacy policy and develop campaigns that leverage the power of image and reputation.

Second, HIE policy makers should establish a broad marketing strategy to enhance patients’ perceptions about the accountability and accuracy of privacy policies, which can foster their trust in the HIE services. Research implications suggest that the HIE initiative managers should consider maximizing the transparency of privacy policy dimensions to induce consumers to read the privacy policy statements and make it a significant consideration in sharing personal information. The findings suggest the importance of educating consumers about the HIE mechanisms and sharing procedures to appeal to their cognitive and emotional trust. As our study shows the significant role of the perceived transparency of privacy policy in building cognitive trust in competence and integrity, a systematic strategy can be performed by health care entities to better demonstrate the dimensions of HIE’s privacy statement. For instance, national educational programs, health conferences, and webinars that are easily accessible to a wide range of people can be administered to clearly publicize the key goals and policies of the national HIE efforts. Educational forums available on official health websites, Web-based tutorials accessible on patient portals or Web-based health communities, and computerized help programs can be used by health care organizations to improve the transparency of HIE efforts, broadcast their privacy policies, and increase public awareness on digital exchange mechanisms.

Third, according to the findings, the lack of public awareness about the expected benefits of HIE and the components of its privacy policy may impede the progress of sharing information between providers because of a lack of patients’ cognitive and emotional trust in the HIE. This study suggests that both physicians and health care organizations (such as hospitals) can directly play an important role in persuading patients to give consent to sharing medical records using HIEs. Physicians’ role may be more effective because they have face-to-face encounters with patients, and during consultations, they can enlighten the patients about the privacy policy of electronic sharing mechanisms. Hospitals can also influence how patients build trust in the HIE by educating them through brochures, leaflets, diagrams, and fact sheets that are comprehensible for an average person. These efforts should be able to clearly highlight why health information is shared, what types of information can be exchanged, how such information is shared from a point to another, what exchange mechanisms are used, who can access the medical data, what security safeguards will protect their records, and how often the transmission takes place.

Fourth, beside the educational programs designed to market expected benefits of the HIE for patients, the HIE administrators and health care organizations should attempt to meet patients’ privacy policy expectations. According to the results, a comprehensive privacy statement that is able to address privacy policy requirements should have 6 related dimensions. The notice component should clearly state the type of health data collected and shared, specify the purposes of data exchange, identify any potential recipients of the data, explain how the shared personal information will be used, and indicate whether the exchange of the requested data is voluntary or required. The choice factor should provide transparent options for patients about how to put a limit on sharing personal information, give patients clear choice by asking for permission before disclosing health information to a third party, and clearly provide individual’s choice of sharing health information under specific conditions (eg, in the case of emergency). The access component should describe whether individuals are able to access their personal information and are able to correct inaccuracies in their personal information and state whether they have the right to delete their personal information from the HIE records. The security feature should clearly state the safeguards used to protect the data from unauthorized access and explain the required technology used to ensure cross-border data protection. The retention factor should clearly state the duration of keeping personal data, describe the time frame during which providers will access shared health information, and explain the reasonable approaches to ensure that the private health data are not kept longer than is necessary. Finally, the enforcement component should be clear enough to describe the actions that will be taken according to the law against who violate the privacy principles and provide a set of guidelines and enforcement mechanisms to assure that information sharing on the Web will abide by privacy laws.

Finally, the results show that the transparency of HIE privacy policies is important for patients, and the contents significantly affect their cognitive trust in the HIE competence and integrity. HIE privacy policies should not be merely prepared to meet legal requirements and protect health care entities from potential privacy lawsuits; most importantly, they should be able to address patients’ privacy and security risks. Evidence suggests that in general, the contents of hospitals’ privacy policies are prepared in a way that are not easily understandable by the majority of adults, and, in turn, patients are not usually interested in reading policy statements [97,98]. Presenting all required dimensions of privacy practices in the privacy policies, standardizing writing patterns, and improving the transparency of contents, along with the simplification of legal jargon, special expressions, and specialized language used to develop policy statements, can help patients better understand their rights and controls of their sensitive health information. HIE privacy statements should choose and focus on the contents that are able to resolve the most pressing privacy concerns. Administrators of the HIE initiatives can modify the contents and elements included in privacy policies based on the issues that rank high on their patients’ concern list. The regulatory agencies can also play an important role by conducting educational workshops or training for the HIE organizations and health care providers on how to develop comprehensive privacy policies and running awareness campaigns to advance public understanding of information privacy and privacy practices of the HIE initiatives. By doing so, legal punishments and privacy violation penalties can be minimized and patients’ trust in the HIE initiatives will be enhanced. Thus, the different dimensions and requirements of the HIE privacy policy point out the importance of health care providers’ endeavor to prepare a detailed privacy policy framework for HIEs. The entities involved in the HIE efforts should also analyze the existing privacy statement’s language, format, and wording to ensure the privacy policy clearly reflects the 6 components. The findings of this research would be useful for the HIE organizations to create robust, accessible, comprehensive, and transparent privacy statements for information exchange purposes to improve patient trust in the HIE initiatives.

### Limitations and Future Research

Our research has some limitations that call for additional studies. The opportunity of further research on the development of patient trust in the HIE is rich, and we urge future studies to continue to test and improve the proposed model, theoretical logics, and hypotheses. The proposed model in this study may serve as a starting point in delineating the formation of patient trust in the HIE, and further research is required to investigate the processes of trust building and its different dimensions, antecedents, and outcomes. Consistent with previous research in other contexts [39], in this study, we separated the cognitive trust in the HIE competence from cognitive trust in the HIE integrity to better demonstrate the different role each dimension may play in the patient’s decision-making process. Future studies can extend this model by measuring cognitive trust as a second-order construct. Moreover, another promising area for future research is to investigate the likely difference between patients’ trust in individual providers and trust in health care organizations with large administrative systems to identify how this difference will influence the degree of cognitive and emotional trust in the HIE technology. Thus, the possible variance of trust transfer mechanism based on provider types can be further examined in future research.

In this study, we leveraged several measures and filters to recruit participants who were familiar with a regional, patient-centered HIE’s functions and its privacy policy. However, as a self-rated sample of participants on MTurk was used, there is a small chance that some individuals were not completely aware of the HIE mechanisms and formed their own mental construal of the IT artifact. Therefore, we suggest that further studies use a different method to ensure that subjects are knowledgeable about the HIE efforts. For instance, future research can recruit informed patients who are directly referred by the providers participating in the HIE initiatives. Moreover, our study used a Web-based survey to recruit participants digitally. Thus, we only considered individuals who accessed the internet and were healthy enough to participate in the Web-based survey. Future studies can use other data collection means and sampling strategies to reach out to a sample that is generalizable to a wide range of health care consumers.

The variance of trust factors (cognitive and emotional) explained by the proposed model was around 50%. This implies that there are other variables that could be included in the model. Future research should examine other factors that may affect the trust-building process in the HIE context. Moreover, the intention to opt in to HIE and willingness to disclose health information may be affected by the sensitivity levels of health information. For instance, if a patient perceives that his or her health information is highly sensitive (eg, mental health information and sexual health diseases), he or she may prefer to hide it from health care providers and, in turn, become less likely to opt in to an HIE network that shares such personal information with other providers. Future studies can measure the possible effect of perceived health information sensitivity on the 2 behavioral intentions. In addition, this study does not focus on a specific group of patients with sensitive information (such as individuals living with HIV). Future research can extend this model to identify how the cognitive and emotional trust will be developed in the context where health information is highly sensitive. Finally, the 2 behavioral intentions may follow a 2-stage model, in which intention to opt in to HIE may occur before the willingness to disclose health information. We recommend that future studies investigate the possible relationship between the 2 behavioral intentions and the impact of these intentions on actual patient behaviors.

### Conclusions

Sharing personal information and dependence on a technology to conduct information exchange are a trust-related behavior. However, how patient trust develops in the HIE context, within which the HIE technology is implemented and used by health care organizations for sharing purposes, has not been clearly discussed yet. To fill this research gap, this study mainly draws upon the trust transfer mechanism to articulate patients’ trust-building process. The cognitive trust in the HIE’s competence and integrity, in addition to the emotional trust, is a fundamental part of patients’ opt-in decisions and willingness to disclose their health information. The findings also show that patients’ initial trust in health care providers and their perception about the role of trusted providers in the development of privacy policy are found significant to determine the opt-in intentions and willingness to share health information in the future. These results can contribute to trust transfer theory and enrich the literature on the HIE efforts. Practitioners can also identify how to leverage the benefit of patients’ trust and trust transfer process to promote the HIE initiatives nationwide.

## Appendix

Multimedia Appendix 1 Online survey.

## Abbreviations

ACP: average congruency percentage
AVE: average variance extracted
e-commerce: electronic commerce
GFI: goodness of fit index
HIE: health information exchange
IS: information system
IT: information technology
MTurk: Mechanical Turk
TRA: Theory of Reasoned Action
VIF: variance inflation factor
