# Supplementary material for: The Impacts of the Perceived Transparency of Privacy Policies and Trust in Providers for Building Trust in Health Information Exchange: Empirical Study
Source: JMIR Med Inform. 2019 Nov 26;7(4):e14050. doi: 10.2196/14050 (PMC6913631; doi:10.2196/14050)
Supplement: Multimedia Appendix 1 [file medinform_v7i4e14050_app1.pdf]

Multimedia Appendix 1 - Online survey

Dear participants,

Please read the provided information about HIE networks and case scenario first, and then answer the following questions accordingly.

Thank you very much for your time.

Health Information exchange (HIE) projects:

This study is designed to evaluate the levels of trust and perceptions of patients who experienced with the Health Information Exchange (HIE) technology. Patients can experience HIE based on their experience with a physician (health care provider) that uses electronic mechanisms to exchange the patient health information with other healthcare organizations.

Sharing health information using HIE is different from offline-based information exchange (such as paper copy of medical records or diagnostic imaging files stored on a CD or memory stick carried by patients themselves). A physician who participates in an HIE network can share the patient's electronic health information (as required) with other health care entities (such as other hospitals, physician practices, laboratories, pharmacies, primary care, and emergency department) in the future using different electronic exchange mechanisms.

A treating physician can share the patient's health information with other health care professionals using various HIE mechanisms (such as the Direct project model or Query-based model). The Direct project model automates point-to-point processes in which a health care provider can directly send patient data electronically to a known recipient (another provider) using a secure network. In the Query-based model (or look-up), patient data are aggregated from multiple healthcare institutions and stored in a central repository. By doing that, physicians can release patients' information to a hub where other physicians can get access to it through a lookup process.

Scenario:

Please think of the last time you have experienced with the HIE technology. For instance, the last time you remember that your treating physicians used an HIE network to share your health information electronically with other health care providers. Now, please answer the following questions based on your last experience:

Have you ever visited a health care provider participating in an HIE network?

- Yes
- No

Please specify the **main reason** that made you become aware of HIE projects:

- I have become aware of HIE through visiting a doctor who participates in an HIE network
- I have become aware of HIE through participating in an HIE network (By using Personal Health Record systems)

- I have become aware of HIE through the internet searching and social media (online information gathering)
- I have become aware of HIE through reading magazines/ newspaper articles or ads
- I have become aware of HIE through my friends and family
- Other (Please specify)

I know that my health information has been exchanged -------- by my health care providers who use HIE systems.

One time

2 - 5 times

6 -10 times

More than 10 times

All following questions are based on 5-point Likert scales with anchors ranging from (1) strongly disagree to (5) strongly agree

Measurement instrument

Construct		Item	Wording
Perceived transparency of privacy policy	Notice	NT1	The HIE has the notice that clearly states type of health data collected and shared
		NT2	The HIE has the notice that clearly states the purposes for which the health data is to be exchanged
		NT3	The HIE has the notice that clearly identifies any potential recipients of the data
		NT4	The HIE has the notice that clearly explains how the shared personal information will be used
		NT5	The HIE has the notice that clearly states whether the exchange of the requested data is voluntary or required
	Choice/ consent	CH1	Privacy statement clearly provides individual's choice of limiting personal information sharing
		CH2	Privacy statement clearly explains individual's consent on personal information disclosure
		CH3	Privacy statement clearly provides choice given to individual to opt-in or opt-out
		CH4	Privacy statement gives users clear choice by asking for permission, before disclosing personal information to third party
		CH5	Privacy statement clearly provides individual' choice of sharing health information under specific conditions (such as in the case of emergency)
	Access	ACC1	Privacy policy describes whether individuals are able to access their personal information
		ACC2	Privacy policy explains whether individuals are able to correct inaccuracies in their personal information
		ACC3	Privacy policy states whether individuals have right to delete their personal information from the HIE record
		ACC4	Privacy policy clarifies whether individuals are allowed to review their shared personal information
	Security	SEC1	Privacy statement clearly states the safeguards used to protect data from unauthorized access
		SEC2	Privacy statement clearly states the required actions to ensure personal data security during information sharing

		SEC3	Privacy statement clearly explains the required technology to ensure cross-border data protection
		SEC4	Privacy statement clearly informs the steps taken to prevent personal information from being disclosed for any unauthorized purposes
	Retention	RET1	Privacy policy clearly states the duration of keeping the personal data
		RET2	Privacy policy clearly explains the time frame that providers will access shared health information
		RET3	Privacy policy clearly states the steps to delete personal data if it is no longer required for the consented purpose
		RET4	Privacy policy clearly explains the reasonable approaches to ensure private health data is not kept longer than is necessary
	Enforcement	ENF1	Privacy statement clearly discloses that there is a law sanctioning those who violate the privacy policy
		ENF2	Privacy statement clearly explains the actions that will be taken according to the law against who violate the privacy principles
		ENF3	Privacy statement clearly provides a set of guidelines and enforcement mechanisms to assure that online information sharing will abide by privacy laws
Cognitive trust in HIE's competency		COM1	The HIE technology is a real expert system in information sharing
		COM2	The HIE project is capable and competent in sharing health information electronically
		COM3	The HIE effort is able to adapt to specific and unforeseen situations
		COM4	The HIE has a standard of competency to carry out information sharing
Cognitive trust in HIE's integrity		INTEG1	I think the HIE system is honest
		INTEG2	I consider the HIE to be of integrity
		INTEG3	Promises made by the HIE are likely to be reliable
		INTEG4	I expect that the HIE system keeps promises it makes
		INTEG5	The HIE does not make false claims and information
Emotional trust in HIE		EMOT1	I feel secure about relying on the HIE for sharing health information
		EMOT2	I feel comfortable about relying on the HIE for information sharing among providers

	EMOT3	I feel content about relying on the HIE for exchanging personal health data
	EMOT4	I feel safe about relying on the HIE to disseminate my sensitive information
Opt-in intention to HIE	INT1	I accept to opt in to HIE to exchange my personal information among health care entities
	INT2	Using HIE is something I would support
	INT3	I would like my health care providers to use HIE to share my personal information
	IN4	I will endorse my physicians to use HIE in their practice
Willingness to disclose health information	WILL1	I am very likely to provide my health information to HIE systems in the future
	WILL2	I am not likely to hide my health information from a provider using HIE
	WILL3	In the future, I am willing to provide personal information to a physician using HIE system
	WILL4	It is probable that I will release my health information to be exchanged through HIE
Trust in healthcare providers	THP1	I believe that any and all parties involved in my health care process: (are not honest at all/are very honest)
	THP2	I believe that any and all parties involved in my health care process: (care about their own interests only/care about their patients all the time)
	THP3	I believe that any and all parties involved in my health care process: (are opportunistic/are dependable)

Gender

- Male
- Female

Age:

Under 20

20 – 29

30 – 39

40 – 49

50 – 59

60 or older

What category best describes your annual household income (USD $)?

- <$25,000
- $25,000-$49,999
- $50,000-$74,999
- $75,000-$99,999
- ≥$100,000

What category best describes your level of education?

- Less than high school
- High school graduate
- Some college
- 2 year degree (Associates degree)
- 4 year degree (Undergrad/bachelors degree)
- Doctorate

Employment status?

- Employed full time
- Employed part time
- Unemployed
- Retired
- Student

Which of the following best describes your race/ethnicity?

- White
- African American
- Hispanic
- Asian
- Other

In general, I believe that the state of my health is:

- Excellent
- Good
- Fair

- Poor
- Very poor

How comfortable you are with using computers:

- Extremely comfortable
- Somewhat comfortable
- Neither comfortable nor uncomfortable
- Somewhat uncomfortable
- Extremely uncomfortable

How comfortable you are with using the internet:

- Extremely comfortable
- Somewhat comfortable
- Neither comfortable nor uncomfortable
- Somewhat uncomfortable
- Extremely uncomfortable

How comfortable you are with using mobile devices or Apps to check your health care information:

- Extremely comfortable
- Somewhat comfortable
- Neither comfortable nor uncomfortable
- Somewhat uncomfortable
- Extremely uncomfortable

How do you rate your computer skills?

- Excellent
- Good
- Average
- Poor
- Very poor

To what extent you are likely to seek greater understanding of your conditions?

- Extremely likely
- Somewhat likely
- Neither likely nor unlikely
- Somewhat unlikely
- Extremely unlikely

To what extent you are likely to connect to your providers in the pursuit of care goals?

- Extremely likely
- Somewhat likely
- Neither likely nor unlikely
- Somewhat unlikely
- Extremely unlikely

Have you ever ...

participated in an online patient community?

- Yes
- No

had an account for a personal health record to maintain and manage your health information?

- Yes
- No
